# Myocardial Regional Interstitial Fibrosis is Associated With Left Intra-Ventricular Dyssynchrony in Patients With Heart Failure: A Cardiovascular Magnetic Resonance Study

**DOI:** 10.1038/srep20711

**Published:** 2016-02-05

**Authors:** Lian-Yu Lin, Cho-Kai Wu, Jyh-Ming Jimmy Juang, Yi-Chih Wang, Mao-Yuan Marine Su, Ling-Ping Lai, Juey-Jen Hwang, Fu-Tien Chiang, Wen-Yih Issac Tseng, Jiunn-Lee Lin

**Affiliations:** 1 Division of Cardiology, Department of Internal Medicine, National Taiwan University College of Medicine and Hospital, Taipei, Taiwan; 2 Department of Medical Imaging, National Taiwan University Hospital, Taipei, Taiwan; 3 Center for Optoelectronic Medicine, National Taiwan University College of Medicine, Taipei, Taiwan

## Abstract

Left ventricular (LV) dyssynchrony is associated with poor prognosis in patients with heart failure (HF). The mechanisms leading to LV dyssynchrony are not fully elucidated. This study evaluates whether myocardium regional variation in interstitial fibrosis is associated with LV dyssynchrony. Forty-two patients with systolic heart failure (SHF), 76 patients with heart failure with preserved ejection fraction (HFpEF) and 20 patients without HF received cardiovascular magnetic resonance imaging (MRI) study. LV was divided into 18 segments by short-axis view. In each segment, regional extracellular volume fraction (ECV) and the time taken to reach minimum regional volume (Tmv) were derived. Intra-LV dyssynchrony were represented by maximum difference (Dysyn_max) and standard deviation (Dysyn_sd) of all Tmv. The results showed that among the covariates, only age (1.87, 95% CI: 0.61–3.13, p = 0.004) and ECV (3.77, 95% CI: 2.72–4.81, p < 0.001) were positively associated with Tmv. The results remained robust in certain subgroups. In conclusion, we demonstrated that LV myocardium regional variation in interstitial fibrosis is closely related to LV intra-ventricular dyssynchrony irrespective of the LV global function. These data might help explain the pathophysiology of LV dyssynchrony and it’s underlying mechanisms leading to poor prognosis.

Non-uniform contraction of left ventricular (LV) myocardial walls due to declining electromechanical synchronicity (LV dyssynchrony) may result in myocardial inefficiency^1^ and is associated with poor prognosis in patients with heart failure (HF)^2^,^3^,^4^. The mechanisms leading to LV dyssynchrony are not fully elucidated but myocardial fibrosis might play some role. In one study, the possibility that myocardial fibrosis, identified by late gadolinium-enhanced cardiac magnetic resonance (LGE-CMR) imaging, might lead to significant LV dyssynchrony has been shown in patients with non-ischemic cardiomyopathy (NICMP)^5^. However, myocardial fibrosis identified by LGE-CMR is obviously not the main mechanism leading to LV dyssynchrony since LV dyssynchrony is also common in many disease entities without late gadolinium enhancement (LGE)^6^,^7^. A critical drawback to the technique of LGE-CMR imaging is that it relies on the difference in signal intensity between scarred and normal adjacent myocardium to generate image contrast. Since collagen deposition in NICMP is commonly diffuse, the technique of LGE-CMR often shows no regional scarring^8^.

Previously, the only methodology available to assess myocardial diffuse interstitial fibrosis was the histopathology assessment of endomyocardial tissue biopsy specimen. Recently, diffuse myocardial interstitial fibrosis has been proved to be able to be quantitatively defined by CMR contrast-enhanced T1 mapping technique^8^,^9^,^10^. For example, Mascherbauer *et al.* showed that CMR contrast-enhanced T1 time is associated with outcome in patients with HF and preserved ejection fraction^9^. In Multi-Ethnic Study of Atherosclerosis (MESA) sub-study, extracellular volume fraction (ECV), an indicator for diffuse myocardial interstitial fibrosis, was demonstrated to be higher in elderly and women^10^. Through this noninvasive technique, diffuse myocardial interstitial fibrosis could be broadly studied in clinical settings.

In the current study, we planned to investigate the relationship between myocardium regional interstitial fibrosis and dyssynchrony both in HF patients with impaired or preserved systolic function as well as in age-matched controls. We hypothesized that regional difference in myocardial interstitial fibrosis might be closely associated with LV dyssynchrony.

## Results

Sequential 42 patients with SHF, 76 patients with HFpEF, and 20 patients without HF were enrolled in the study. The demographics of the study population were summarized in Table 1. There was no significant difference in age among groups, but there were more male patients in SHF group compared to HFpEF (78.6% vs. 51.3%, p = 0.005) and non-HF (78.6% vs. 30.0%, p < 0.001) groups. The prevalence of wide QRS (≧120 ms) was also higher in patients with SHF than that in HFpEF and non-HF controls (28.6% vs. 11.8% and 0.0% respectively). In the HF group, patients with SHF had a higher rate of prior myocardial infarction (40.5% vs. 18.4%, p = 0.015) than patients with HFpEF, whereas patients with HFpEF had a higher rate of hypertension than patients with SHF (75% vs. 35.7%, p < 0.001). The prevalence of prior myocardial infarction was also higher in HFpEF group than that in non-HF group (18.5% vs. 0.0%, p = 0.037). Around half of the patients with HF had CAD (47.6% for SHF and 50.0% for HFpEF) and patients with SHF had higher prevalence of dilated cardiomyopathy (14.3% for SHF and 1.3% for HFpEF).

The MRI parameters of the three groups were shown in Table 2. The chance of LGE was higher in patients with SHF than that in HFpEF (69.0% vs. 34.2%, p < 0.001). LV volume and mass indices including EDV, ESV, LVM, EDVi, ESVi and LVMi were all significantly higher in the SHF group as compared to that in HFpEF and non-HF groups. Among these indices, only LVM (118.5 gm; IQR: 91.6–150.8 gm vs. 90.0; IQR: 75.7–107.8 gm, p = 0.003) and LVMi (66.8 gm; IQR: 56.5–84.5 gm vs. 54.5; IQR: 49.4–59.7 gm, p = 0.001) were significantly different between HFpEF and non-HF groups. For myocardial diffuse fibrosis, patients with SHF had significantly higher mean ECV than patients with HFpEF (35.5%; IQR: 30.2–37.7% vs. 29.6%; IQR: 26.7–32.6%, p < 0.001) and without HF (35.5%; IQR: 30.2–37.7% vs. 28.0%; IQR: 25.6–29.3%, p < 0.001). The mean ECV was also significantly higher in patients with HFpEF than that in patients without HF (29.6%; IQR: 26.7–32.6% vs. 28.0%; IQR: 25.6–29.3%, p = 0.028). For dyssynchrony indices, the dysyn_max was higher in patients with SHF than that in patients with HFpEF (400.0 ms; IQR: 285.9–509.2 ms vs. 185.6 ms; IQR: 160.0–239.7 ms, p < 0.001) and without HF (400.0 ms; IQR: 285.9–509.2 ms vs. 175.0 ms; IQR: 151.2–206.7 ms, p < 0.001). Also the dysn_sd was higher in SHF group than that in HFpEF (112.9 ms; IQR: 78.1–141.4 ms vs. 55.7 ms; IQR: 43.5–74.6 ms, p < 0.001) and non-HF(112.9 ms; IQR: 78.1–141.4 ms vs. 48.4 ms; IQR: 44.3–54.5 ms, p < 0.001) groups. The dyssyn_sd was also higher in HFpEF than that in non-HF (55.7 ms; IQR: 43.5–74.6 ms vs. 48.4 ms; IQR: 44.3–54.5 ms, p = 0.030) patients.

**Figure 1:**
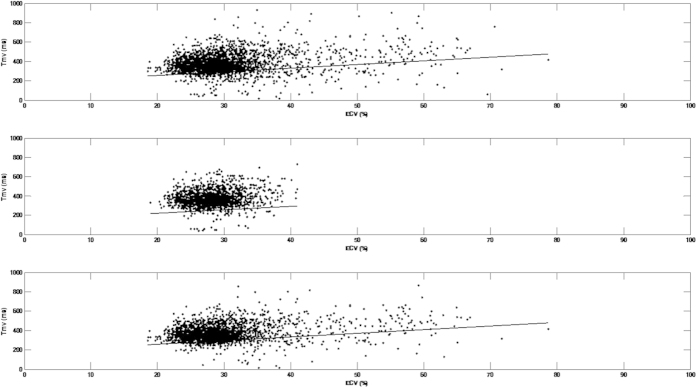
Scatterplot of extracellular volume fraction (ECV) and time taken to reach minimum regional volume (Tmv) of each segment in all patients (Fig. 1A), patients without late gadolinium enhancement (LGE) (Fig. 1B) and patients with QRS <120 ms (Fig. 1C). The black line is a regression line indicates the fixed effect of ECV on Tmv.

The results of mixed model analyses were shown in Table 3. Model selection analysis by Bayesian information criterion (BIC) suggested that the random effects of intercept and ECV should be considered in the final model. As demonstrated in Table 3, among the covariates, only age (1.87, 95%CI: 0.61–3.13, p = 0.004) and ECV (3.77, 95%CI: 2.72–4.81, p < 0.001) were positively associated with Tmv. Also shown in Table 3, the results remained similar if we only included patients without LGE and wide QRS. Both age and ECV were positively associated with Tmv. This indicated that 1% increase of ECV was associated with 3.56 to 3.77 ms increment of Tmv. The interaction term LGE x ECV was not significant suggesting that the presence of LGE did not modify the effect of ECV on Tmv. Figure 1 was the scatterplot of ECV and Tmv of each segment in all patients (Fig. 1A), patients without LGE (Fig. 1B) and with QRS <120 ms (Fig. 1C). The fixed effect of ECV on Tmv was also demonstrated by the regression lines in Fig. 1.

## Discussion

To our knowledge, this is the first clinical study to investigate the association between regional interstitial fibrosis and LV intra-ventricular dyssynchrony. Our result demonstrates that regional interstitial fibrosis is positively associated with LV intra-ventricular dyssynchrony irrespective of the LV global function.

In physiological condition, the fibrillary collagen network is in intimate contact with myocardial cells and plays a critical role in the maintenance of ventricular shape and function^7^. When pathological processes take effect, the activation of myofibroblasts and the increase in collagen synthesis result in two major types of fibrosis, reactive interstitial fibrosis and replacement fibrosis, commonly affect diastole first and subsequently involve systolic performance^11^. Reactive fibrosis or interstitial fibrosis has mostly been described in hypertension, DM, aging heart and idiopathic dilated cardiomyopathy where the activation of renin-angiotensin aldosterone system, beta-adrenergic system and the excess of reactive oxygen species are major contributors. Replacement fibrosis corresponds to the replacement of myocytes after cell necrosis by plexiform fibrosis and is mostly seen in patients with previous myocardial infarction or myocarditis^7^. Even though LGE-CMR is a sensitive and reproducible method to detect and quantify replacement fibrosis but it’s sensitivity is limited for the assessment of diffuse interstitial fibrosis. For example, in our study, the ECV levels of the so called remote zone are higher in patients with SHF than in HFpEF and control groups. These remote areas are considered as normal myocardium in LGE-CMR but are actually abnormal in T1 mapping.

The recent advancement in acquisition sequences enables us to quantify diffuse myocardial interstitial fibrosis by T1 mapping with high spatial resolution. Several studies have used post-contrast myocardial T1 time to quantify diffuse myocardial interstitial fibrosis in patients with various cardiac diseases^8^,^9^,^10^. However, post-contrast myocardial T1 time is affected by several factors such as magnetic field strength, the timing of post-contrast MOLLI acquisition, the type of MOLLI scheme, the amount of contrast injected and renal function of patients^12^. In contrast, myocardial ECV is measured by normalization of myocardial T1 time with blood T1 time which is more stable and less affected by these factors^13^.

The association between LV dyssynchrony and fibrosis has been reported before. In one study, a higher global fibrosis index, defined as more LV segments with LGE, is associated with a higher degree of LV dyssynchrony in patients with NICMP^5^. In the current study, instead of using a global fibrosis index, we investigated the correlation between LV regional interstitial fibrosis and dyssynchrony and found that they were intimately related. Since collagen deposition in NICMP is commonly diffuse, this finding has revealed one possible mechanism leading to LV dyssynchrony in NICMP without LGE. Even though the causal relationship could not be established, it is possible that the two factors might be reinforced with each other forming a vicious cycle. In one hand, regional variation in interstitial fibrosis could lead to dyssynchrony. Myocardial fibrosis might have adverse effects on myocyte excitability, cell-to-cell coupling and impaired intracellular and intercellular calcium handling, all of which are potential mechanisms leading to dyssynchrony^14^. Human study also showed that presence of LGE in patients with newly diagnosed dilated cardiomyopathy is associated with subsequent deterioration in LV dyssynchrony^15^. On the other hand, dyssynchrony itself might cause regional variation in interstitial fibrosis. In one canine model, LV dysynchrony by RV apical pacing could cause a higher stretch in late-activated myocardium leading to regional variation in extracellular matrix remodeling^16^, protein expression^17^ and possibly fibrosis.

Myocardium interstitial fibrosis and dyssynchrony have been shown to coexist in early stage of many disease entities such as DM, hypertension and in aging process^18^,^19^,^20^,^21^,^22^,^23^. In patients with SHF and HFpEF, interstitial fibrosis and dyssynchrony also have been demonstrated to be associated with severity of heart function and prognosis^2^,^3^,^4^,^8^,^9^,^10^,^24^. Our study further demonstrated that these two factors are closely related. All these imply that the LV regional interstitial fibrosis might potentially be a one of the common pathways leading to LV dyssynchrony.

Our findings might have some important clinical implications. For example, previous study has demonstrated that patients receiving cardiac resynchronization therapy (CRT) have worse prognosis if there are scars in the regions where the LV pacing leads are placed to^25^. On the other hand, placement of LV leads in the latest activated myocardial segments are associated with better survival^25^. Since myocardium with normal appearance on LGE images might actually have high degree of extracellular fibrosis^26^, the latest activated myocardial segments without LGE might have high degree of diffuse fibrosis which might preclude the effect of CRT. From this point of view, MRI study combined LGE and T1 imaging might be necessary to guide optimal lead placement.

Finally, in order to investigate the relationship between regional ECV and dyssynchrony index, the image modality used to measure LV dyssynchrony should be interrogated with T1 mapping. Thus, we used cine MRI instead of cardiac ultrasonography to quantify LV dyssynchrony. In general, myocardial velocity timing could be evaluated by ultrasound tissue Doppler imaging (TDI) and myocardial strain timing could be evaluated by MRI tagging and 2D-strain echocardiography (2DSE)^27^,^28^. However, TDI and 2DSE are limited by the acoustic windows and the anisotropic sensitivity of the method, while MRI tagging requires extensive computation and shows a low sensitivity for reduced wall thickening^27^. Since all these methods estimate the contraction timing, we used cine MRI to derive the contraction timing of the mass center of a specific segment in radial direction. This technique is similar to a previous validated method based on cine MRI^27^.

## Conclusions

In conclusion, we demonstrated that LV myocardium regional variation in interstitial fibrosis is a major determinant of LV intra-ventricular dyssynchrony irrespective of the LV global function. These data might help explain the pathophysiology of LV dyssynchrony and it’s underlying mechanisms leading to poor prognosis.

### Study limitations

There are several limitations of the study. First, this study design was cross-sectional, we could not clarify the causal relationship between regional interstitial fibrosis and dyssynchrony. Second, our study has no histological evidence to validate the results regarding the changes of myocardial ECV because all subjects have no indication for endomyocaridal biopsy. Third, the temporal resolution of cine MRI is inferior to echocardiography. This results in lower sensitivity of the MRI-derived LV dyssynchrony index, only subjects with higher degree of dyssynchrony could be identified.

## Methods

### Ethics Statement

The research was approved by the institutional review board of the National Taiwan University Hospital Ethics Committee. The study was conducted in accordance with the approved guidelines. All study participants provided written informed consent.

### Patient populations

One hundred and eighteen patients who met the following criteria were enrolled as the HF group: 1) they had HF symptoms of New York Heart Association (NYHA) classification functional Class II to III or a history of HF symptoms/signs by Framingham criteria^29^, and 2) the symptoms and signs of HF persisted for more than 3 months. From the HF group, seventy six patients with left ventricular ejection fraction (LVEF) above 45% and with LV diastolic dysfunction documented by tissue Doppler echocardiography, defined as the mean of septal and lateral mitral annular early diastolic velocity (Ea) <8 cm/sec^30^, were assigned to the HF with preserved ejection fraction (HFpEF) group. Forty patients with EF below 45% were defined as the systolic heart failure (SHF) group. Twenty patients without a history of symptoms/signs were recruited as a non-HF group. Subjects were excluded from the study if they had significant valvular heart diseases indicated for percutaneous or surgical intervention, chronic atrial fibrillation, chronic pulmonary disease, active myocardial ischemia defined by a positive stress test or un-revascularized significant (70%) stenosis in coronary arteries by angiography, or estimated glomerular filtration rate (GFR) <30 mL/min/1.73 m2.

### Imaging Acquisition

CMRI was performed on a 3 T MRI system (Trio, Siemens, Erlangen, Germany) with an 8-channel cardiovascular phased array torso coil. Myocardial T1 mapping was performed with an ECG-triggered Modified Look Locker Inversion recovery (MOLLI) sequence before and 10 minutes after a 0.15 mmole/kg intravenous administration of the gadolinium-based contrast agent (Omniscan, Winthrop Laboratories, GE, NJ). The MOLLI protocol used two Look-Locker cycles to acquire 7 images over 11 heart beats, the scanning parameters were TR/TE, 1.9 ms/1.0 ms; flip angle, 35°; minimum inversion time, 110 ms; inversion time increment, 80 ms; matrix size, 256 × 192; slice thickness, 6 mm; spatial resolution, 1.28 mm; GRAPPA acceleration factor, 2; number of inversions, 2; images acquired after first inversion, 5; pause 4 heart beats and images acquired after second inversion, 2. Five evenly-spaced short-axis slices were acquired sequentially from the LV base to apex. After post-contrast T1 acquisition, LGE images were acquired using an ECG-triggered phase-sensitive inversion recovery (PSIR) prepared segmented fast gradient echo pulse sequence^31^ at the same short-axis slices as those in the myocardial T1 mapping to identify the focal fibrosis or scaring.

Cine MRI was performed using a segmented balanced steady-state gradient echo pulse sequence with a retrospective ECG R-wave trigger. The scanning parameters were TR/TE, 3.0 ms/1.5 ms; flip angle, 46°; matrix size, 256 × 208 and spatial resolution, 1.21 mm. Multiple short-axis slices were prescribed from the mitral orifice to LV apex with slice thickness of 8 mm and gap of 2 mm. The true temporal resolution was 63 ms and thirty cardiac phases were reconstructed retrospectively for each slice level.

### Imaging Analysis

Three slices of short-axis LV images at the basal, papillary muscle and apical levels were chosen for further analyses. The cine and T1 MRI were interrogated by choosing the nearest locations at the z axis. At each level, the LV myocardium was divided into 6 segments and in each segment, the mean ECV value and the wall motion were calculated as followings. For T1 MRI, the regions of interest (ROIs) in the blood of central cavity and each segment of LV myocardium were drawn for each slice. The averaged T1 values of the segmented ROIs were then computed. After subtracting the pre-contrast values from the post-contrast values, the changes of the relaxation rate (1/T1) in the blood and in the myocardium were obtained. Myocardial ECV values were calculated using the ratio of the change in relaxation rate in the myocardium to that in the blood and multiplied by (1- hematocrit)^32^.

For LV function and mass analysis, endocardial and epicardial contours of the LV were determined at each slice level on cine MRI and the area enclosed by each contour was computed^33^. LV volumes for each time point were then determined by the Simpson’s rule to obtain the volume-time curve of the LV. End-diastolic volume (EDV) and LV end-systolic volume (ESV) of the LV were assessed from the volume–time curve for the maximal and minimal values and were used to compute LVEF. LV mass was computed as the difference between LV epicardial volume at end-diastole and EDV, multiplied by the density of the myocardium, 1.05 g/cc. LV volumes and mass indexed to body surface area (BSA) were also measured from EDV (EDVi), ESV (ESVi) and LVM (LVMi) divided by BSA. For wall motion analyses, the mass center of each segment was defined and the radial motion of the center was traced in the cine MRI during the cardiac cycle. The time from ECG R-wave to minimum regional volume of each LV segment was defined as time taken to reach minimum regional volume (Tmv). Intra-LV dyssynchrony were represented by maximum difference (Dysyn_max) and standard deviation (Dysyn_sd) of all Tmv. Image analysis was performed using software developed in-house provided by Matlab 7.9 (Mathworks, Inc., Natick, MA, USA).

### Statistical analysis

Since Shapiro-Wilk test showed that some of the variables were not normally distributed, variables among groups were compared by non-parametric methods. Continuous variables were expressed as medians and interquartile ranges (IQR) and categorical variables were expressed as percentages. Categorical variables were compared among different groups of patients by using Chi-square tests. Continuous variables were tested by the nonparametric Kruskal-Wallis test and the Mann-Whitney U test was used for post-hoc analysis for comparison of the medians between different groups. To test the association between LV dysynchrony and myocardial interstitial fibrosis in all subjects, a linear mixed model using repeated-measures analysis was constructed to analyze the relationship between Tmv and ECV in each LV segment since we collected 18 segments of Tmv and ECV in one patient. Since age, ECV and Tmv were normally distributed, the model was adjusted for age, gender, presence of LGE, QRS over 120 ms, history of diabetes mellitus (DM), hypertension, coronary artery disease (CAD), LV mass index and grouping. Also, an interaction term LGE x ECV was added to test whether the presence of LGE modifies the effect of ECV on Tmv. BIC was used to determine whether the random effects of intercept and ECV should be considered in the model or not. A value of p < 0.05 was considered significant. Statistical analyses were performed using the SPSS software package, version 19 (SPSS, Chicago, IL, USA).

## Additional Information

**How to cite this article**: Lin, L.-Y. *et al.* Myocardial Regional Interstitial Fibrosis is Associated With Left Intra-Ventricular Dyssynchrony in Patients With Heart Failure: A Cardiovascular Magnetic Resonance Study. *Sci. Rep.* **6**, 20711; doi: 10.1038/srep20711 (2016).

## Figures and Tables

**Table 1:** Basic demographics of the studied subjects.

**Patient characteristics**	**SHF (n = 42)**	**HFpEF (n = 76)**	**non-HF (n = 20)**
Age, year	64.5 (58.5~73.3)	67.5 (60.0~75.0)	64.5 (60~77)
Gender (male)%	78.6*^†^	51.3	30.0
BSA, m^2^	1.70 (1.62~1.82)	1.72 (1.59~1.85)	1.66 (1.57~1.79)
QRS ≧ 120 ms, %	28.6*^†^	11.8	0.0
**Comorbidities**			
HTN, %	35.7*^†^	75.0	80.0
DM, %	26.2	31.6	20.0
Dyslipidemia, %	45.2	47.4	60.0
CKD, %	9.5	5.3	5.0
Stroke, %	2.4	2.6	5.0
MI, %	40.5*^†^	18.4^†^	0
PAOD, %	4.8	3.9	5.0
**Etiologies for HF**			
CAD, %	47.6^†^	50.0^†^	20.0
Dilated CMP, %	14.3*^†^	1.3	0.0

Abbreviations: SHF, systolic heart failure; HFpEF, heart failure with preserved ejection fraction; non-HF, patients without heart failure; BSA, body surface area; HTN, hypertension; DM, diabetes mellitus; CKD, chronic kidney disease; MI, myocardial infarction; PAOD, peripheral arterial occlusive disease; CAD, coronary artery disease; HF: heart failure, CMP, cardiomyopathy

*p < 0.05 compared with HFpEF.

^†^p < 0.05 compared with the non-HF group.

**Table 2:** LV function and mass for patients with/without heart failure.

	**SHF (n = 42)**	**HFpEF (n = 76)**	**Non-HF (n = 20)**
LGE, %	69.0%*^†^	34.2%^†^	0.0%
EDV, ml	173.6 (134.2~226.1)*^†^	92.0 (72.2~115.0)	87.9 (68.9~102.6)
ESV, ml	113.3 (85.0~149.8)*^†^	20.3 (13.6~38.6)	17.8 (10.5~26.4)
EDVi, ml/m2	104.0 (73.5~128.9)*^†^	52.9 (43.9~65.2)	48.5 (42.9~60.3)
ESVi, ml/m2	70.9 (45.3~90.1)*^†^	12.2 (8.0~24.0)	10.1 (6.9~14.5)
EF, %	34.3 (26.6~40.4)*^†^	77.6 (62.7~82.0)	79.8 (73.9~83.9)
LVM, gm	150.0 (126.8~182.6)*^†^	118.5 (91.6~150.8)^†^	90.0 (75.7~107.8)
LVMi, gm/m2	90.9 (73.7~101.7)*^†^	66.8 (56.5~84.5)^†^	54.5 (49.4~59.7)
Mean ECV, %	35.5 (30.2~37.7)*^†^	29.6 (26.7~32.6)^†^	28.0 (25.6~29.3)
Dyssyn_max, ms	400.0 (285.9~509.2)*^†^	185.6 (160.0~239.7)	175.0 (151.2~206.7)
Dyssyn_sd, ms	112.9 (78.1~141.4)*^†^	55.7 (43.5~74.6)^†^	48.4 (44.3~54.5)

Abbreviations: SHF, systolic heart failure; HFpEF, heart failure with preserved ejection fraction; non-HF, patients without heart failure; LGE, late gadolinium enhancement; EDV, end-diastolic volume; ESV, end-systolic volume; EDVi, end-diastolic volume index; ESVi, end-systolic volume index; EF, ejection fraction; LVM, left ventricular mass; LVMi, left ventricular mass index; ECV, extracellular volume fraction; Dysyn_max, maximum difference of time taken to reach minimum regional volume of all myocardial segments. Dysyn_sd, standard deviation of time taken to reach minimum regional volume of all myocardial segments.

*p < 0.05 compared with HFpEF.

^†^p < 0.05 compared with the non-HF group.

**Table 3:** Mixed model analysis by using time taken to reach minimum regional volume as dependent variable.

	**ALL (n = 138)**		**Patients without LGE (n = 88)**		**Patients with QRS <120 ms (n = 117)**	
Age	1.87 (0.61~3.13)	0.004	1.82 (0.53~3.11)	0.006	1.26 (0.32~2.21)	0.009
Sex (M vs. F)	8.07 (−21.41~37.58)	0.588	2.07 (−25.43~29.57)	0.881	−2.51 (−25.65~20.64)	0.830
LGE	6.02 (−67.82~79.87)	0.873	N/A	N/A	5.55 (−59.14~70.25)	0.866
QRS >120 ms	18.70 (−17.84~55.25)	0.313	9.98 (−31.36~51.32)	0.632	N/A	N/A
Diabetes mellitus	−15.17 (−42.91~12.56)	0.281	6.51 (−20.51~33.54)	0.632	−4.03 (−25.26~17.21)	0.708
Hypertension	15.61 (−14.71~45.92)	0.310	−10.15 (−44.38~24.09)	0.556	−3.59 (−28.27~21.09)	0.774
CAD	11.32 (−16.81~39.45)	0.427	3.84 (−24.57~32.24)	0.788	−6.91 (−29.11~15.29)	0.538
LV mass index	0.07 (−0.61~0.47)	0.793	0.45 (−0.12~1.02)	0.117	0.03 (−0.47~0.41)	0.888
Group						
SHF vs. Control	4.00 (−46.83~54.82)	0.876	40.89 (−13.28~95.06)	0.137	14.02 (−24.32~52.35)	0.470
HFpEF vs. Control	18.55 (−20.60~59.69)	0.369	18.95 (−11.16~49.06)	0.213	11.02 (−16.79~38.83)	0.433

ECV	3.77 (2.72~4.81)	<0.001	3.56 (1.75~5.37)	<0.001	3.76 (2.64~4.89)	<0.001
LGE x ECV	8.45 (−219.62~236.53)	0.942	N/A		7.51 (−205.69~220.71)	0.945

Abbreviations: LGE, late gadolinium enhancement; CAD, coronary artery disease; SHF, systolic heart failure; HFpEF, heart failure with preserved ejection fraction; ECV, extracellular volume.
